# Maternal dietary components in the development of gestational diabetes mellitus: a systematic review of observational studies to timely promotion of health

**DOI:** 10.1186/s12937-023-00846-9

**Published:** 2023-03-07

**Authors:** Victoria Lambert, Sonia Edith Muñoz, Carla Gil, María Dolores Román

**Affiliations:** 1 Instituto de Investigaciones en Ciencias de la Salud, Consejo Nacional de Investigaciones Científicas y Técnicas. Universidad Nacional de Córdoba, Córdoba, Argentina; 2 Escuela de Nutrición, Facultad de Ciencias Médicas, Universidad Nacional de Córdoba, Córdoba, Argentina

**Keywords:** Pregnancy, Diet, Health promotion, Gestational diabetes mellitus

## Abstract

**Background:**

There is ample evidence that considers diet as an important factor in the prevention of gestational diabetes mellitus (GDM). The aim of this review is to synthesise the existing evidence on the relationship between GDM and maternal dietary components.

**Methods:**

We performed a systematic bibliographic search in Medline, Latin American and Caribbean Health Sciences Literature (Lilacs) and the Latin American Nutrition Archive (ALAN) of regional and local literature, limiting the searches to observational studies published between 2016 and 2022. Search terms related to nutrients, foods, dietary patterns and the relationship to GDM risk were used. The review included 44 articles, 12 of which were from America. The articles considered different topics about maternal dietary components as follows: 14 are about nutrient intake, 8 about food intake, 4 combined nutrient and food analysis and 18 about dietary patterns.

**Results:**

Iron, processed meat and a low carbohydrate diet were positively associated with GDM. Antioxidant nutrients, folic acid, fruits, vegetables, legumes and eggs were negatively associated with GDM. Generally, western dietary patterns increase GDM risk, and prudent dietary patterns or plant-based diets decrease the risk.

**Conclusions:**

Diet is considered one of the causes of GDM. However, there is no homogeneity in how people eat nor in how researchers assess diet in different contextual conditions of the world.

**Supplementary Information:**

The online version contains supplementary material available at 10.1186/s12937-023-00846-9.

## Introduction

Gestational Diabetes Mellitus (GDM) is one of the most common complications that occur during pregnancy [1], it affects approximately 5–17% of pregnancies worldwide and is becoming a public health problem due to the great burden of the disease and its increasing prevalence [2]. GDM can be defined as the alteration of glucose tolerance of variable severity that begins or is recognized for the first time during the current pregnancy [3, 4]. Generally, this resolves when the pregnancy ends, but it makes the woman prone to the development of premature labour, caesarean sections, hypertensive disorders, a new development of GDM in subsequent pregnancies, obesity and metabolic syndrome, and an increased risk of developing type 2 diabetes and cardiovascular diseases in the years following her pregnancy [3–6]. On the other hand, babies born to mothers with GDM are at increased risk of developing foetal hyperinsulinemia, neonatal hypoglycaemia, jaundice, being large for their gestational age, and developing obesity and type 2 diabetes later in life thus generating a cycle that favours metabolic dysfunction through the generations [6–8].

In the aetiology of GDM, various factors are identified that interact in a complex causal network. It is known that maternal age, pre-pregnancy overweight and obesity, excessive weight gain during pregnancy, sedentary lifestyle are risk factors for its development [9, 10]. Recently, the association between diet and GDM has been studied, but the evidence is still unclear. It is particularly noteworthy that diet before and during pregnancy is a potentially modifiable factor that can modulate the risk of GDM [11–15]. Likewise, it has been evidenced this pathology has a significant economic impact in all countries, health systems and individuals, especially those with low incomes [3, 4]. The available evidence on the diet-GDM relationship is still scarce in the major world regions [15–17]. Hence, the objective of this review is to synthesise the evidence between nutrients, food, dietary patterns and other features of diet and the risk to develop GDM considering regional differences in eating habits.

## Methods

### Search strategy

A systematic review was conducted following the Preferred Reporting Items for Systematic Reviews and Meta-Analyses (PRISMA) 2020 statement, an updated guideline for reporting systematic reviews. In the search we introduced terms referring to the relationship between components of the mother’s diet and GDM. We performed a systematic bibliographic search in MEDLINE and The Cochrane library for international publications, and Latin American and Caribbean Health Sciences Literature (Lilacs) or the Latin American Nutrition Archive (ALAN) for regional and local literature, limiting the searches to observational studies published since 2016.

### Search terminology

The search terms included Medical Subject Headings (MESH) terms and keywords as “diabetes, gestational” AND “diet, western”, “feeding behavior”, “diet”, “food”, “food industry”, “food and beverages”, “eating”, “energy intake”, “nutrients”, “diet records”, “dietary pattern”, “maternal diet”, “food frequency questionnaire”, “zinc”, “mineral”, “vitamin”, “nutrition”, “fruits”, “vegetables”, “vitamin pattern”, “dietary intake”, “flavonoids”, “antioxidant”, “iron” OR “meat”, “fiber” OR “fibre”, “fat” OR “fatty acids”, “micronutrients” OR “macronutrients”, “carotenoid” OR “vitamin A” OR “carotene”, “vitamin C” OR “vitamin D” OR “folate” OR “vitamin b2” OR “vitamin b6”, “calcium” OR “potassium”. The search was limited to human observational studies published up to December 2022. Reference lists from relevant articles and reviews were manually searched for potentially relevant citations not detected by the electronic search.

### Selection criteria and data extraction

The selected studies met the following inclusion criteria: full text and original study; observational study design like cohort, case-control, cross-sectional in women of reproductive age; and studies whose objectives, methodological designs and results included the association between maternal dietary components before or during pregnancy and development of GDM.

Studies reporting on dietary supplements were excluded. Studies reporting on abnormal glucose tolerance but not on GDM, review papers, conference abstracts and intervention studies were not included. Studies examining eating disorders, perceptions, sensations, clinical trials and qualitative methodological studies were not considered. Titles, abstracts and full-text articles identified from the literature search were screened for eligibility against inclusion and exclusion criteria.

Data were extracted for the evaluation of the study from the authors, year of publication, study design, number of women, number of GDM cases, recruitment location and period, baseline age, exclusion criteria, dietary factors and assessment method, screening method and diagnostic criteria. Finally, information on the results of the study was extracted: mean, SD, SE, OR, or 95% CI of maternal dietary components together with the number of women in each group, effect estimates, and 95% CIs for associations between dietary factors and GDM and confounding factors used in the analyses.

### Risk of bias and quality assessment

Quality assessment of the studies included was independently performed by two researchers and discrepancies were resolved by discussion with a third reviewer. The Newcastle-Ottawa Scale was used to evaluate the quality of assessment of exposure and outcome variables of interest [18]. View Supplementary Information.

### Data synthesis and analysis

Search results indicating the significance and direction of the associations observed were qualitatively summarised in tables for each maternal dietary component by study design. Information on study characteristics was extracted to describe studies and populations.

## Results

### Study selection

**Fig. 1:**
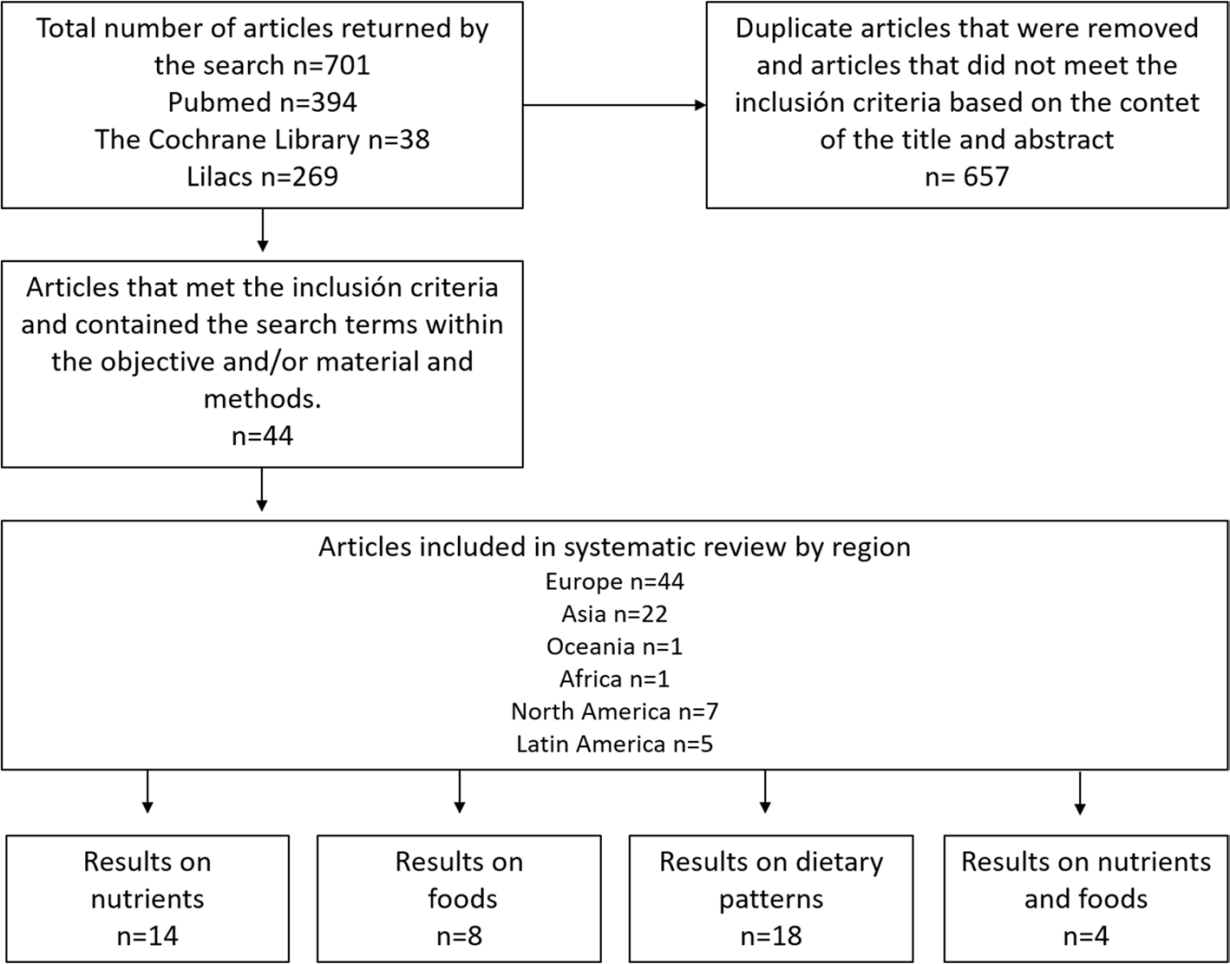
Flow chart with selection criteria of articles included in the systematic review on the association between dietary factors and GDM at the international and regional levels

The selection process of the articles included in the review is summarised in Fig. 1. At first, according to the search terms entered, the search returned a total of 701 articles, of which we found 394 in PubMed, 38 in The Cochrane Library and 269 in Lilacs. A total of 657 duplicate articles and articles that did not meet the inclusion criteria based on title and abstract were removed. Finally, the total number of articles included in the review was reduced to 44, 5 were published in Latin America, 7 in North America, 8 in Europe, 22 in Asia, 1 in Africa and 1 in Oceania. The maternal dietary components considered were macro or micronutrients intake (14 articles), food intake (8 articles), 4 articles combined nutrient and food analysis, and 18 dietary patterns. Of all the articles from Latin America, 2 deal with macro-and micronutrient intakes, 1 with food intake and 2 articles with dietary patterns. Most studies addressing food from a dietary pattern approach come from East Asia.

### Study characteristics

Study characteristics, including a number of subjects, study type, population characteristics, maternal dietary component analysis and its effect on GDM are described in Tables 1, 2, 3, 4 and 5 according to a regional location. Results were mostly prospective cohort studies (26 studies), 13 were case-control studies and 5 were cross-sectional. The majority of the cross-sectional studies were Latin American. All the reports included women aged between 19 and 45 years old who visited hospitals or healthcare centres. The maternal dietary components were analysed before and during pregnancy in 3 articles, 9 articles before pregnancy and 32 articles during pregnancy.

**Table 1:** Summary of results from Asian observational studies on diet and gestational diabetes

Paper	Regional location	Number of subjects	Study type	Population characteristics	Maternal dietary component	Effects of diet on GD
Asadi M, et al. 2019 [19]	Iran- Asia	n:278 cases:130 controls:148	case-control	women aged 19–40 years who came from six healthcare centres between 2014 and 2015	pre-pregnancy dietary pattern	Prudent dietary pattern: Odds Ratio (OR) = 0.88, 95% Confidence Interval (CI): 0.44–0.99, p-trend = 0,02
Sedaghat F, et al. 2017 [20]	Iran- Asia	n: 388 cases:122 controls:266	case-control	women aged 18–40 years who visited major general hospitals 2009–2010	pre-pregnancy dietary pattern	Western dietary pattern: OR = 1.97, 95% CI: 1.27–3,04
Lamyian M, et al. 2017 [21]	Iran- Asia	n: 1026	cohort	women aged 18–45 years who visited 5 universities of medical sciences’ hospitals 2010–2011	pre-pregnancy fast food consumption	Total fast food consumption: OR = 2.12 95% CI: 1.12–5.43, p-trend = 0.03; french fries: OR = 2.18 95% CI: 1.05–4.70, p-trend = 0.12
Zamani B. et al. 2019 [22]	Iran- Asia	n:460 cases:200 controls:260	case-control	women aged 22–44 years who visited the nutrition clinic in AL-Zahra and Shahid Beheshti hospitals, Isfahan. Gestational age between 25 and 28 weeks	pregnancy plant-based diet (PDI)	Higher PDI score: OR = 0.47; 95% CI: 0.28–0.78, *P* = 0.004
Zareei S. et al. 2018 [23]	Iran - Asia	n: 204 cases:104 controls:100	case-control	women who visited the maternity ward of Valiasr Hospital in Fasa Town, 2016. No data about gestational age	pregnancy dietary pattern	Unhealthy dietary pattern: OR = 2.838 95% CI: 1.039–7.751, *p*-value: 0.042; healthy dietary pattern: OR = 0.284,95% CI:0.096–0.838, *p*-value: 0.023
Du HY et al. 2017 [24]	China - Asia	n: 753	cohort	women who visited the Maternal and Child Health Care Hospital in China, 2013–2014. Gestational age between 5 and 15 weeks.	pregnancy dietary pattern	Western pattern: OR = 4.40, 95% CI: 1.58–12.22, p-trend: 0.004); traditional pattern: OR = 4.88, 95% CI: 1.79–13.32, p-trend: 0.002)
Chen Q. et al. 2020 [25]	China - Asia	n: 9556 cases:1464 controls:8092	case-control	women who visited the First Affiliated Hospital of Shanxi Medical University in China, 2012–2016.	pre-pregnancy and pregnancy dietary pattern	Vegetable pattern 1 year prior to conception OR = 0.94; 95% CI: 0.89–0.99, p-trend: 0,025; first trimester of pregnancy OR = 0.94; 95% CI: 0.89–0.99, p-trend: 0,018; second trimester of pregnancy OR = 0.91; 95% CI: 0.86–0.96, p-trend: < 0.001
Zhou X et al. 2018 [26]	China - Asia	n:2755	cohort	pregnant women from the Tongji Maternal and Child Health Cohort. They have visited the maternity clinic in one of three public hospitals in Wuhan, China, since 2013–2016. Gestational age between 8 and 16 weeks.	pregnancy dietary pattern	High Fish-meat-eggs scores (OR for quartile 4 vs. quartile 1 = 1.83; 95% CI 1.21, 2.79; *p* = 0·007); high rice-wheat-fruits scores (OR for quartile 3 v. quartile 1 = 0.54; 95% CI 0.36, 0.83; *p* = 0.010)
Dong H et al. 2021 [27]	China - Asia	n: 1455	cohort	women > 12 weeks gestations who visited the Sichuan Provincial Hospital for Women and Children, Southwest China, 2017	pregnancy dietary pattern	Overall Low carbohydrate dietary (LCD) pattern RR = 1.24, 95% CI 1.01–1.52, *p* = 0.026
Chen Q. et al. 2019 [28]	China, Asia	n: 9556 cases:1464 controls:8092	case-control	women who visited the First Affiliated Hospital of Shanxi Medical University in China, 2013–2016.	pre-pregnancy and pregnancy vitamin dietary pattern	Vitamin dietary pattern 1 year prior to conception: OR = 0.90; 95% CI: 0.85–0.95, p-trend: < 0.0001; first trimester of pregnancy OR = 0.90; 95% CI: 0.86–0.95, p-trend: < 0.0001; second trimester of pregnancy OR = 0.90; 95% CI: 0.85–0.95, p-trend: < 0.0001
Liu C et al. 2020 [29]	China - Asia	n: 3009	cohort	women who visited the First Affiliated Hospital of Shanxi Medical University in China, 2013–2016. Gestational age < 16 weeks.	pregnancy vitamin C intake	Above adequate dietary vitamin C intake OR = 0.68, 95% CI: 0.49–0.95.
Saraf-Bank S et al. 2018 [30]	Iran - Asia	n: 463 cases:200 controls:263	case-control	pregnant women aged 22–44 years who visited the Nutrition Clinic of Isfahan and Shahid Beheshti Hospital as well as Azzahra Hospital. Gestational age between 5 and 28 weeks.	dietary acid intake	Highest tertile of potential renal acid load (PRAL): OR = 9.27; 95% CI: 4.00–21.46, p-trend: < 0,001
Parast VM et al. 2017 [31]	Iran - Asia	n: 80 cases:40 controls:40	case-control	pregnant women who visited the Department of Obstetrics and Gynecology of the Shahid Beheshti Hospital, 2016. Gestational age between 24 and 28 weeks	antioxidant nutrients intake	Total capacity antioxidant (TAC): OR 9.6; 95% CI: 3.4–26.8); *p* value: < 0.001; intakes of vitamin E OR = 1.5; 95% CI: 1.2–1.9; *p* value: < 0.001; intakes of selenium OR = 8.2; 95% CI: 1.3–52.0; *p* value: 0.026; intakes of zinc OR = 1.7; 95% CI: 1.2–2.5; *p* value: < 0.001
Gao Q et al. 2019 [32]	China- Asia	n: 1978	cohort	pregnant women with maternal age > 18 years, who visited three public hospitals in Wuhan, 2013–2016. Gestational age between 8 and 16 weeks.	carotenoids and lycopene intake	Highest quartile of lycopene intake OR = 0.50; 95% CI 0.29, 0.86; p-trend = 0·007)
Kyozuka H et al. 2021 [33]	Japan - Asia	n: 92764	cohort	Japanese women from the Japan Environment and Children’s Study (JECS), 2011–2014.	pre-pregnancy antioxidant nutrients intake	Quintil 5 Selenium intake OR = 1.15, 95% CI: 1.01–1.30; quintil 1 Se intake: OR: 1.19, 95% CI: 1.01–1.41
Daneshzad E et al. 2020 [34]	Iran - Asia	n: 463 cases:200 controls:263	case-control	pregnant women aged 22–44 years who visited the Nutrition Clinic of Isfahan, Iran. Gestational age between 25 and 28 weeks.	antioxidants and Vitamin C intake	3 tertil of FRAP (ferric reducing ability of plasma) OR = 0.26, 95% CI: 0.16–0.42; *p* = < 0.0001
Aljanahi A et al. 2020 [35]	Saudi Arabia - Asia	n: 121 cases:72 controls:49	case-control	pregnant women aged 19–45 years who visited the King Fahad University Hospital, Maternal and Children Hospital and Family Medicine of Imam Abdulrahman Bin Faisal University. No data about gestational age.	vitamin D intake, dairy products and eggs consumption	Vitamin D dietary intake is higher among controls compared to cases (*p*-value: 0.021); vitamin C and eggs intake is higher among cases compared to controls (*p* = 0.004; *p* = 0.040); fortified orange juice OR = 3.2; 95% CI: 1.2–8.8, *p*-value: 0.026; fortified yogurt OR = 3; 95% CI: 1.1–8.6, *p*-value: 0.04; low-fat milk OR = 3.2; 95% CI 1.3–7.7; *p*-value 0.01; full-fat milk OR = 0.4; 95% CI: 0.2–0.8; *p*-value: 0.017
Li H, et al. 2021 [36]	China - Asia	n: 2987	cohort	women with a median age of 28.5 ± 3.6 years old. They were from Clinic of the General Hospital of Chinese People’s Armed Police Forces, 2013–2014. Gestational age between 13 and 28 weeks.	fruits, vegetable and fruit juice intake during pregnancy	No association with total fruit and vegetable consumption. A higher quantity of grape, melon, potatoes and fruit juice were positively associated with GDM. A higher quantity of apple, orange and potatoes were negatively associated with GDM (*p* < 0,05).
Yong HY, et al. 2021 [37]	Malaysia - Asia	n: 452	cohort	Women from three maternal child health (MCH) clinics. No data about age	pre-pregnancy and pregnancy consumption of beverage	higher fruit juice intake before pregnancy: AOR = 0.98, 95% CI = 0.97–0.99. In the first trimester: AOR = 0.92, 95% CI = 0.89–0.98) A higher intake of cultured-milk drinks before pregnancy: AOR = 1.03, 95% CI = 1.01–1.08. In the first trimester: AOR = 1.07, 95% CI = 1.02–1.12.
Liu YH et al. 2022 [38]	China - Asia	cases: 143 controls: 345	case-control	no data available	pregnancy dietary patterns	Dietary pattern 2: OR = 2.96, 95% CI: 0.939–9.356, *P* = 0.004
Wang H et al. 2021 [39]	China - Asia	n: 2099	cohort	pregnant women were part of the participants in the Tongji Maternal and Child Health Cohort (TMCHC) study, 2013–2016. Gestational age between 8 and 16 weeks.	pregnant plant-based diet index (PDI)	Highest quartile of PDI: OR 0.43; 95% CI 0.24, 0.77; *p* = 0.005
Zhang X et al. 2021 [40]	China- Asia	n: 9317	cohort	women from public hospitals with obstetric services in South China, 2014–2017.	pre-pregnancy and pregnancy dietary glycemic index, glycemic load and fiber intake	Highest tertile respect to lowest tertile Glycemic index pre-pregnancy: OR 1.12 (95% CI 1.03, 1.19) *p* = 0.01 1st trimester: OR 1.25 (95% CI 1.20, 1.33) *p* = 0.008 2nd trimester: OR 1.29 (95% CI 1.21, 1.48) *p* = 0.005 Glycemic load pre-pregnancy: OR 1.15 (95% CI 1.08, 1.23) *p* = 0.02 1st trimester: OR 1.23 (95% CI 1.10, 1.45) *p* = 0.01 2nd trimester: OR 1.25 (95% CI 1.11, 1.40) *p* = 0.01 Fiber intake pre-pregnancy: OR 0.89 (95% CI 0.83, 0.94) *p* = 0.03 1st trimester: OR 0.83 (95% CI 0.75, 0.91) *p* = 0.01 2nd trimester: OR 0.82 (95% CI 0.73, 0.91) *p* = 0.01

**Table 2:** Summary of results from European observational studies on diet and gestational diabetes

Paper	Regional location	Number of subjects	Study type	Population characteristics	Maternal dietary component	Effects of diet on GD
Tryggvadottir EA, et al. 2016 [41]	Iceland - Europa	n:168	cohort	women aged 18 to 40 years who visited the Prenatal Diagnosis Unit at the National University Hospital, 2012–2013. Gestational age between 19 and 24 weeks.	pregnancy dietary pattern	Prudent dietary pattern: Odds Ratio = 0.54; 95% Confidence Interval (CI): 0.30, 0.98; Prudent pattern in overweight/obese before pregnancy: OR = 0.38; 95% CI 0.18–0.83
Kozlowska A, et al. 2018 [42]	Polish- Europa	n:113	cross-sectional	women > 20 weeks of gestation who visited the Medical University of Warsaw, 2016–2018.	vitamin and mineral pregnancy dietary intake	Mean vitamin C intake was higher in controls than among cases (*p*-value: 0.04). Mean calcium intake was higher in controls than among cases (*p*-value: 0.01)
Bartáková V, et al. 2018 [43]	Czech Republic - Europe	n:363 cases:293 controls:70	case-control	women aged 29–36 years who visited the Diabetes Centre of the University Hospital Brno. Gestational age between 24 and 30 weeks.	pregnancy food intake	Dairy products OR = 3.149; 95% CI: 1.180–8.403, *p*-value: 0.022; goodies OR = 7.600; 95% CI: 0.996–57.964, *p*-value: 0.050; sweet beverages OR = 10.510; 95% CI: 1.395–79.173, *p*-value: 0.022
Donazar-Ezcurra M, et al.2017 [44]	Spanish - Europe	n: 3455	cohort	women prevenient of The SUN project cohort, 2013–2015	pre-pregnancy dietary patterns	Western dietary pattern: OR = 1,56; 95% CI 1,00- 2,43
Mari Sanchis A et al. 2018 [45]	Spanish - Europe	n: 3298	cohort	women prevenient of The SUN project cohort, 2012–2014	pre-pregnancy meat and iron intake	Total meat consumption: OR = 1.67; 95% CI 1.06–2.63; p-trend 0.010; red meat consumption: OR = 2.37; 95% CI 1.49–3.78: p-trend< 0.001; processed meat consumption: OR = 2.01; 95% CI 1.26–3.21; p-trend 0.003
Petry CJ, et al. 2019 [46]	United Kingdom- Europe	n: 865	cohort	pregnant women of 12 weeks of gestation. Cambridge Baby Growth Study (CBGS) recruits.	eggs consumption	Eggs consumption was negatively associated with GDM (*p* = 0.03)
Nicolì F, et al. 2021 [47]	Italy - Europe	n: 376	cohort	Women from the Diabetes Clinic of the University Hospital of Pisa, 2019. No data about age or gestational age	Consumption of non- nutritive-sweetened soft drinks	Non-nutritive-sweetened soft drinks intake: OR 1.814; 95% CI: 1.145–2.874; *p* = 0.011
Yuste Gomez A et al. 2022 [48]	Spain - Europe	n: 103	cohort	women over 16 years old from La Paz University Hospital, no data available about follow-up time. Gestational age < 16 weeks.	pregnancy food intake	Differences in white bread consumption among pregnant women who develop GDM and controls (*p* = 0,012)

**Table 3:** Summary of results from North American observational studies on diet and gestational diabetes

Paper	Regional location	Number of subjects	Study type	Population characteristics	Maternal dietary component	Effects of diet on GD
Bao W et al. 2017 [49]	USA- - North America	n: 15225	cohort	pregnant women aged 24–44 years from the Nurses’ Health Study II cohort, 1991–2001	pre pregnancy vitamin D intake	No association
Li M. et al. 2019 [50]	USA - North America	n: 14553	cohort	pregnant women aged 24–44 years from the Nurses’ Health Study II, 1991–2001.	pre pregnancy food folate intake	Adequate total folate intake (‡400 mg/day) RR = 0.83; 95% CI 0.72–0,95, *p* = 0.007)
Shin D. et al. 2015 [10]	USA- North América	n:253	cohort	pregnant women (16–41 years) included in the National Health and Nutrition Examination Survey (NHANES) 2003–2012. Gestational age of 20 weeks.	pregnancy dietary patterns	“High refined grains, fats, oils and fruit juice” pattern: OR = 4.9; 95% CI 1.4–17.0, p-trend: 0.007; “high nuts, seeds, fat and soybean; low milk and cheese” pattern: OR = 7.5; 95% CI 1.8–32.3, p-trend: 0.009; “high added sugar and organ meats; low fruits, vegetables and seafood” pattern: OR = 22.3; 95% CI 3.9–127.4, p-trend: < 0.0001
Osorio-Yáñez Citlalli et al. 2017 [51]	USA - North America	n: 3414	cohort	pregnant women < 20 weeks of gestation who attending prenatal care clinics affiliated with the Swedish Medical Center and Tacoma General Hospital in Seattle and Tacoma, 1996–2008	calcium and dairy products intake	Calcium intake: RR = 0·58; 95% CI 0·38–0·90; *p* = 0·015; low fat dairy product RR = 0,57; 95% CI: 0,32–1,02 *p* = 0,032; whole grains RR: 0,61; 95% CI: 0,39- 0,95, *P* = 0·019
Darling AM et al. 2016 [52]	USA, Canada - North America	n: 7229	cohort	pregnant women from the Slone Epidemiology Center Birth Defects Study, in the United States and Canada, 1998–2008	pre-pregnancy iron intake	Preconceptional dietary heme-iron 2.53; 95% CI: 1.70–3.78, p-trend: 0.02; preconceptional dietary non-heme iron OR = 0.53; 95% CI: 0.34–0.83, p-trend: 0.13
Chen Z et al. 2021 [53]	United States - America	n: 14926	cohort	Women from the Nurses’ Health Study II, 1991–2001	prepregnancy Plant-based diet index (PDI)	PDI: Q5 compared with Q1: RR 0.70 95% CI 0.56–0.87 ptrend = 0.0004. hPDI: the RR 0.75 95% CI 0.59–0.94, ptrend = 0.009. uPDI was not associated
Lindsay KL, et al. 2022 [54]	United States - America	n: 7997	cohort	women > 13 years old who came from eight U.S. medical centers between 2010 and 2013.	pregnancy and prepregnancy alternative healthy eating index (pAHEI) - 2010	higher adherence to an alternative healthy index (pAHEI): aOR = 0.986 95% CI = 0.973–0.998 *p* = 0.022

**Table 4:** Summary of results from African and Oceanian observational studies on diet and gestational diabetes

Paper	Regional location	Number of subjects	Study type	Population characteristics	Maternal dietary component	Effects of diet on GD
Looman M et al. 2019 [55]	Australia - Oceania	n:3607	cohort	women aged 25–30 years from the prospective Australian Longitudinal Study on Women’s Health cohort, 2003–2015.	pre-pregnancy dietary micronutrient adequacy	Highest quartile of the Micronutrient Adequacy Ratio: RR = 0.61, 95% CI 0.43–0.86, p-trend 0.01.
Mahjoub F et al. 2021 [56]	Tunisia - Africa	n: 120 cases:60 controls:60	case-control	pregnant women aged 26–37 years from the National Institute of Nutrition, 2018. Gestational age between 24 and 32 weeks.	nutrient intake and adherence to a Mediterranean diet during pregnancy	Vitamin D intake: OR = 0.29 [0.15–0.54], *P* < 10–3)

**Table 5:** Summary of results from Latinamerican observational studies on diet and gestational diabetes

Paper	Regional location	Number of subjects	Study type	Population characteristics	Maternal dietary component	Efectos sobre resultados maternos
Sartorelli DS et al. 2019 [57]	Brazil - Latin America	n: 785	cross-sectional	women aged ≥20 years, pre-pregnancy body mass index (BMI) ≥ 20 kg/m2 recruited in five laboratories, 2011–2012. Gestational age between 24 to 39 weeks.	pregnancy minimally processed foods and ultra-processed foods intake	No association
Sartorelli DS et al. 2019 [57]	Brazil - Latin America	n: 785	cross-sectional	women aged ≥20 years, pre-pregnancy body mass index (BMI) ≥ 20 kg/m2 recruited in five laboratories, 2011–2012. Gestational age between 24 to 39 weeks	pregnancy dietary patterns	Dietary pattern 1 (high rice, beans, and vegetables, with low full-fat dairy products, biscuits, and sweets) Odds Ratio (OR) = 0.58; 95% Confindece Intervale (CI) 0.36–0.95; *p* = 0.03
Balbi M et al. 2019 [58]	Brazil - Latin America	n: 785	cross-sectional	women aged ≥20 years, pre-pregnancy body mass index (BMI) ≥ 20 kg/m2 recruited in five laboratories, 2011–2012. Gestational age between 24 to 39 weeks	pregnancy flavonoids intake	No association
Nascimento GR et al. 2016 [59]	Brazil - Latin America	n: 838	cohort	pregnant women from a prenatal health care clinic at the Instituto de Medicina Integral Prof. Fernando Figueira (IMIP), 2011–2014. Gestational age between 15 to 20 weeks.	pregnancy dietary patterns	No association
Barbieri P. et al. 2016 [60]	Brazil - Latin America	n: 799	cross-sectional	pregnant women > 24 weeks of gestation who receiving care at the Public Health System of Ribeirao Preto (SP), Brazil, 2011–2012.	pregnancy dietary fat quality	∑n-3 Polyunsaturated fatty acids intake (PUFA) intake: OR = 0.21; 95% CI 0.08–0.56, *p* = 0,002; α-linolenic intake: OR: 0.15; 95% CI: 0.05–0.42, *p* = < 0.0001; PUFA intake: OR = 0.45; 95% CI: 0.24–0.85, *p* = 0.04

### Quality assessment

The quality assessment ratings and scores of the studies included were carried out according to the Newcastle – Ottawa quality assessment Scale (NOS). Two researchers evaluated quality studies and a third reviewer resolved discrepancies. The Newcastle-Ottawa Scale was adapted to specifically evaluate the quality of exposure and outcome variables of our interest**.** View Supplementary information.

### Association between maternal dietary components and GDM

Some reports have suggested that pre-pregnancy nutritional status and weight gain during pregnancy can modulate the development of GDM [6, 17]. In recent years, diet and healthy nutrition were priorities to prevent adverse events in maternal and child health by the Global Health Alliance in Preconception, Pregnancy and Postpartum (HiPPP) [61]. Increasing evidence suggests that an unbalanced pre-pregnancy and pregnancy diet can have a substantial impact on the health outcomes of women and children and the effects of foetal nutrition may persist into adulthood, with possible intergenerational effects [62–64]. Likewise, various international studies have confirmed the existence of an association between some components of the diet and the incidence of GDM [11, 65]. Below we describe the results obtained on the various ways of studying the components of the diet associated with the risk of developing GDM.

### Association between nutrients and GDM

#### Energy intake

Some authors support the idea that the development of GDM is not caused by dietary nutrients but by the excess of energy [6, 63], because energy intake is the main determinant of gestational weight gain [17]. Thus, Daneshzad E et al. (2020) found that total energy intake was higher in women with GDM than in women without the condition (*P* < 0.05) [34]. Tryggvadottir EA et al. (2015), who studied the GDM-energy relationship from the dietary pattern perspective in 168 pregnant women, reported that those women with obesity ingested more daily energy (2206 ± 535 kcal) than those with overweight (2108 ± 459 kcal) and with normal weight (2160 ± 400 kcal) although energy intake was not associated with GDM [41].

#### Macronutrients

**F**ive reports address the relationship between GDM and carbohydrate, fibre, protein and fatty acid intake. Daneshzad E et al. 2020 show lower intakes of carbohydrates in women with GDM with respect to women without GDM (*p* < 0.05) [34]. A study conducted in China analysed pre-pregnancy and pregnancy dietary glycemic index, glycemic load and fibre intake. Highest tertile respect to lowest tertile glycemic index and glycemic load were protective regarding GDM risk, while fibre intake was promotive (*p* < 0.05) [40]. On the other hand, Zhou X et al. 2018, showed that high fish-meat-eggs scores, which were positively related to protein intake and inversely related to carbohydrate intake, were in turn associated with a higher risk of GDM [OR for quartile (Q) 4 v. quartile (Q) 1: 1.83; 95% CI 1.21, 2.79; P trend = 0.007]. In contrast, high rice-wheat-fruits scores, which were positively related to carbohydrate intake and inversely related to protein intake, were associated with a lower risk of GDM (adjusted OR for Q3 vs Q1: 0.54; 95% CI 0.36, 0.83; *P* trend = 0.010) [26].

With regard to fatty acids, Barbieri P. et al. 2016 found an inverse association between the highest intakes of total n-3 fatty acid, acid alpha-linolenic acid, and GDM [60]. Similarly, in a case-control study in Tunez [56], monounsaturated fatty acids and saturated fatty acids consumptions were significantly higher in the control group (2.3 ± 0.8 vs 1.7 ± 0.7, *p* < 0,05).

#### Micronutrients

Many studies examine the association between dietary micronutrients and adverse maternal outcomes but only some of them evaluate their relationship with GDM. Chen Q. et al. 2019 showed that the “vitamin” pattern (characterised as the consumption of a diet rich in vitamin A, carotene, vitamin B2, vitamin B6, vitamin C, dietary fibre, folate, calcium, and potassium) was positively associated with GDM. For every 25% of the increase in the vitamin factor score during 1 year prior to conception and the first trimester, the GDM risk decreased by 9% (OR: 0.91, 95%CI: 0.86–0.96) and by 10% (OR: 0.90, 95%CI: 0.85–0.95) during the second trimester [28]. In this sense, in another study, women in the highest quartile of the prepregnancy micronutrient adequacy ratio (constructed by vitamin A, folate, niacin, riboflavin, thiamin, vitamin C, vitamin E, calcium, iron, potassium, zinc, phosphorus and magnesium) had a 39% lower risk of developing GDM compared to women in the lowest quartile (RR 0.61, 95% CI 0.43–0.86, p for trend 0.01) [55]. On the other side, micronutrients in isolation were analysed. Folic acid, antioxidant nutrients, calcium and Vit D showed a protective effect. Besides that, iron showed a promoter effect and evidence of selenium was inconsistent.

### Protective micronutrients: folic acid, antioxidants, calcium and vitamin D

Evidence on folic acid intake and GDM varies in the literature. One work showed that pre-pregnancy food folate intake was not associated with GDM risk (P trend = 0.66) while an inverse association was found between GDM and pre-pregnancy total supplement and food folate intake [50].

The association between dietary components with antioxidant action and the development of GDM has been studied to a greater extent than other nutrients. Vitamin C consumption could have a protective effect against GDM. A cohort study showed that pregnant women with dietary vitamin C intake above the recommended level (more than 200 mg/day) experienced lower odds of GDM (OR 0.68, 95% CI: 0.49–0.95) than those with just an adequate intake (115–200 mg/day) [29]. A cross-sectional study observed that the mean vitamin C intake was significantly higher in the control group than in women with GDM [42]. Furthermore, a case-control study observed that intakes of vitamin C, vitamins B6 and A, selenium, and manganese were significantly lower in women with GDM (*P* < 0.05) [34]. In the same way, other studies analysed vitamin E, selenium, zinc, magnesium, potassium, lycopene and flavonoids intake. A case-control study showed consumption of vitamin E (*p* < 0.001), selenium (*p* < 0.05) and zinc (*p* < 0.001) were significantly lower in women with GDM as compared to healthy pregnant women [31]. Moreover, a cohort study found that women with lycopene intake in the highest quartile reduced 5% the risk of GDM (OR 0·50; 95% CI 0·29, 0·86; P for trend = 0·007) compared with the lowest quartile [32]. Also, a cohort study observed a high prevalence of inadequate dietary micronutrient consumption for magnesium (52.5%), potassium (63.8%) and vitamin E in pregnant women (78.6%), however, it was not associated with the risk of GDM [55]. Nor did a cross-sectional study find any association between flavonoids intake and GDM but it showed a very low intake of flavonoids in pregnant women [58].

One cross-sectional, two cohort and two case-control studies evaluated a protective effect of calcium and Vitamin D intake against GDM too. Another cross-sectional study found the mean calcium intake was significantly higher in the control group than among the cases [42]. One cohort study showed that, although not significantly, calcium intake was inversely associated with the risk of GDM (RR = 0·58; 95% CI 0·38, 0·90; *P* = 0·015). Besides, in those women who consumed less than 1200 mg/day, increasing dietary intake by 200 mg/day reduced the risk of GDM by 22% (RR = 0.78; 95% CI: 0.61–0.99; *p* value = 0.042) [51]. The other cohort found dietary vitamin D intake and total supplement and dietary vitamin D intake were inversely associated with risk of developing GDM, although it was not significant [49]. The last two case-control studies, when compared in terms of intake, women with GDM presented lower intake of vitamin D in relation to the controls (2.3 ± 2.1 μg / j vs. 6.3 ± 3.3 μg / j, *P* < 10–3) [35, 56].

### Promoter micronutrients: iron and selenium

Regarding to promoter micronutrients, two cohort studies positively associated pre-pregnancy heme iron intake with GDM (OR = 2.21 95% CI 1.37–3.58, p-trend 0.003) [45] (OR 1.55; 95% CI 0.98, 2.46) [49]. On the other hand, preconception dietary non-heme iron was associated with a decreased risk of GDM (OR: 0.48; 95% CI 0.28, 0.81) [52]. As regards to selenium, a cohort study showed that pregnant women with intakes in the highest quintile (OR: 1.15, 95% CI: 1.01–1.30) and also those in the lowest one presented increased risks of GDM (OR: 1.19, 95% CI: 1.01–1.41), using quintile 3 as the reference [33].

### Food and other dietary features

Three case-control studies, six cohorts and one cross-sectional study found an association between food or meals and the risk of developing GDM. The case-control studies evaluated adherence to dietary acid load (calculated using several nutrient intakes such as phosphorus, protein, calcium, magnesium and potassium) and the mediterranean diet (adherence to vegetables, fruits, legumes, cereals and bread, pasta, rice; fish and seafood; meat, poultry; dairy products; alcohol and ratio MUFAs/SFAs), food consumption and the asociation with GDM risk [30, 43, 60]. Women with higher scores of dietary acid load and a low mediterranean diet score were more likely to have GDM during pregnancy (OR = 9,27; 95% CI: 4.00–21.46) [30, 56]**.** Also, women with GDM exhibited significantly more frequent poultry, pork and smoked meat, dairy products and sweet beverages consumption. Women with GDM consumed less fresh vegetables compared to controls [43]. Another cohort study shows a positive association between higher quantities of grape, melon, and fruit juice and GDM, and a negative association between higher quantities of apple, orange and potatoes (*p* < 0,05) [36]. Two cohort studies found an association between risk of GDM, egg and fast food consumption [21, 46]. .A negative association was shown between the frequency of egg consumption and GDM [46]. On the other hand, total fast-food (OR 2.12; 95% CI 1.12–5.43) and french fries consumption (OR 2.18; 95% CI 1.05–4.70) was associated with higher risk of GDM [21]. In the last cohort study, a difference in white bread consumption between women with and without GDM was found (*p* = 0,012) [48] Finally, the cross-sectional study assessed the association between risk of GDM and the intake of minimally processed and ultra-processed foods in Brazilian women, but no association was found [66]. Women with GDM were consuming more eggs (*p* = 0 .040). It was also found that full-fat milk was negatively associated with GDM and low-fat milk, fortified yoghurt, and fortified orange juice were positively associated with GDM (*p* < 0.05) [35]. Regarding the beverage intake, a higher fruit juice intake before pregnancy (AOR = 0.98, 95% CI = 0.97–0.99) and in the first trimester (AOR = 0.92, 95% CI = 0.89–0.98) had a lower GDM risk. On the other hand, a higher non-nutritive-sweetened soft drinks intake (OR 1.814; 95% CI: 1.145–2.874; *p* = 0.011) [37], a higher intake of cultured-milk drinks before pregnancy (AOR = 1.03, 95% CI = 1.01–1.08) and during first trimester (AOR = 1.07, 95% CI = 1.02–1.12) had an increased GDM risk [47].

### Prepregnancy and pregnancy dietary patterns

Analysing a diet, the dietary pattern approach allows combining different dietary components (nutrients, foods, food groups) into a single measure of dietary exposure. It provides information about the nature, quality, quantity, proportions and frequency of consumption of different foods and beverages that are dominant in an individual’s diet [67, 68]. Dietary patterns can be influenced by food availability and socio-cultural factors [69]; therefore, it is worth analysing their regional variations because, principally in Asia, two different dietary patterns, prudent and western, during pre-pregnancy and pregnancy and GDM risk were described in the literature.

First, two case-control studies and two cohort study evaluated the association between pre pregnancy dietary patterns and GDM. Asadi et al. 2019 identified that prudent dietary pattern (higher intakes of fruits, low-fat dairy, potato, egg, fish, poultry, nuts, organs meat and red meat) was inversely associated with GDM risk (OR = 0.88, 95% CI: 0.44–0.99), and the western dietary pattern (higher intakes of sugar-sweetened beverages, refined grain products, fast foods, salty snacks, sweets and biscuit, mayonnaise and saturated oils) was significantly associated with GDM risk [19]. Unlike these findings, Sedaghat F, et al. 2017 found an association between western dietary pattern (high in sweets, jams, mayonnaise, soft drinks, salty snacks, solid fat, high-fat dairy products, potatoes, organ meat, eggs, red meat, processed foods, tea, and coffee) and GDM before and after adjustment for confounders (OR = 1.97, 95% CI: 1.27–3.04, OR = 1.68, 95% CI: 1.04–2.27), but they did not find a significant association of GDM with the prudent pattern (higher intake of liquid oils, legumes, nuts and seeds, fruits and dried fruits, fish and poultry whole, and refined grains) and risk of GDM [20]. In the same way, in a cohort study Donazar-Ezcurra M, et al. 2017 identified two prepregnancy dietary patterns, a western dietary pattern (high consumption of meat-based products and processed foods) and the Mediterranean dietary pattern (high consumption of vegetables, fruits, fish and non-processed foods), similar to Iranian prudent patterns. They found a positive association in the multivariable model between the highest quartile of adherence to western dietary pattern and GDM compared with the lowest quartile (OR 1·56; 95% CI 1·00, 2·43), however they did not find an association between the Mediterranean dietary pattern and GDM incidence (OR 1·08; 95% CI 0·68, 1·70) for the highest quartile compared with the lowest [44].

Second, two cohort and two case-control studies evaluated prepregnancy and pregnancy dietary patterns and GDM risk association in Asia. Chinese women with adherence to a vegetable dietary pattern (consumption of green leafy vegetables, cabbages, carrots, tomatoes, eggplants, potatoes, mushrooms, peppers, bamboo shoots, agarics, and garlic and bean products) prior to conception (OR 0.94; 95% CI, 0.89 to 0.99), during the first trimester (OR, 0.94; 95% CI, 0.88 to 0.99) and during the second trimester of pregnancy (OR, 0.91; 95% CI, 0.86 to 0.96) lowered the GDM risk [25]. In the same sample, it was determined that the adherence to a vitamin-nutrient pattern (high intake of dietary vitamin A, carotene, vitamin B2, vitamin B6, vitamin C, dietary fibre, folate, calcium, and potassium) 1 year prior to conception (OR: 0.91, 95%CI: 0.86–0.96), in the first trimester (OR: 0.91, 95%CI: 0.86–0.96) and the second trimester of pregnancy (OR: 0.90, 95%CI: 0.85–0.95) decreased GDM risk [28]. Also, a higher adherence to a plant-based diet index in North America, decreased GDM risk (OR 0.43; 95% CI 0.24, 0.77; *p* = 0.005) [53]. In the same way an association between higher adherence to an alternative healthy index (pAHEI) and lower GDM risk was found (aOR = 0.986 95% CI = 0.973–0.998 *p* = 0.022) [54].

Lastly, adherence to a pregnancy dietary pattern and its association with GDM risk was a bit more studied than the pre-pregnancy dietary pattern. Three case-control studies and eight cohort studies were found. In the Iranian case-control studies, a plant-based diet index (PDI), and a healthy and unhealthy dietary pattern were identified. Zamani B. et al. 2019 showed that adherence to a high plant-based diet index score was inversely associated with risk of GDM (OR = 0.47; 95% CI: 0.28–0.78, *P* = 0.004) [22]. An unhealthy dietary pattern (high intake of mayonnaise, soda, pizza and sugar) was associated with GDM (OR = 2.838,95% CI:1.039–7.751), and the adherence to a healthy dietary pattern (high intake of leafy green vegetables, fruits, poultry and fish) in the Q4 had 149% higher chance not to develop GDM (OR = 0.284,95% CI:0.096–0.838) compared with the Q1 [23]. Similar results were found in a study that analysed association between overall PDI, healthy PDI and GDM risk in North America (RR 0.70 95%CI 0.56–0.87 *p* = 0.0004; RR 0.75 95% CI 0.59–0.94 *p* = 0.009) during 2010–2013 [39]. In this sense, a European cohort study identified the prudent dietary pattern (positive factor loadings for seafood; eggs, vegetables, fruits and berries, vegetable oils, nuts and seeds, pasta, breakfast cereals, and coffee, tea and cocoa powder, and negative factor loadings for soft drinks and french fries) was associated with a lower risk of GDM (OR: 0.54; 95% CI: 0.30, 0.98), even if they included only overweight and obese women (OR: 0.31; 95% CI: 0.13, 0.75) [41].

In a USA cohort study, three dietary patterns associated with increased risk for GDM were identified, the “high refined grains, fats, oils and fruit juice” pattern (AOR 4.9; 95% CI 1.4–17.0), “high nuts, seeds, fat and soybean; low milk and cheese” pattern (AOR 7.5; 95% CI 1.8–32.3) and the “high added sugar and organ meats; low fruits, vegetables and seafood” pattern (AOR 22.3; 95% CI 3.9–127.4) [10].

In China, Zhou X et al. 2018 showed that adherence to high fish–meat–eggs scores, which were positively related to protein intake and inversely related to carbohydrate intake, were associated with a higher risk of GDM (OR for Q4 v. Q1: 1·83; 95% CI 1·21, 2·79; Ptrend = 0·007). On the other hand, high rice-wheat–fruits scores, which were positively related to carbohydrate intake and inversely related to protein intake, were associated with a lower risk of GDM (OR for Q3 v. Q1: 0.54; 95% CI 0.36, 0.83; P trend = 0.010) [26]. In this sense, another cohort study found the adherence to a low carbohydrate diet (< 70 g/day) with high consumption of animal protein was associated with GDM risk [27]. Also In China, Du HY et al. 2017 identified four dietary patterns. Compared with the prudent pattern, the Western pattern and the traditional pattern were associated with an increased risk of GDM (OR = 4.40, 95% CI: 1.58–12.22; OR = 4.88, 95% CI: 1.79–13.32). Compared to the lowest quartile, Q3 of the western pattern scores and Q3-Q4 of the traditional pattern scores were associated with a higher risk of GDM [24]. Another study conducted by Liu YH et al. 2022 found relationship between homocysteine-related dietary patterns (positive factor loadings for wheaten food, livestock meat, eggs and negative factor loading for coarse cereals, green leafy vegetables, dried fungi and algae, milk group and nuts) and higher GDM risk (OR = 2.96, 95% CI: 0.939–9.356, *P* = 0.004) [38]. In the last two studies realised in Brazil, Nascimento GR et al. 2016 did not find an association between dietary patterns during early pregnancy and GDM [59], but Sartorelli DS et al. 2019 showed dietary pattern 1 (high rice, beans, and vegetables, with low full-fat dairy products, biscuits, and sweets) was inversely associated with GDM (OR 0.58; 95% CI 0.36–0.95; *p* = 0.03) [57].

Pre-pregnancy and pregnancy dietary patterns characterised by fruits, vegetables, whole grains, fish and dairy products had a protective effect against GDM risk. A dietary pattern characterised by refined grains, sugar, fats, meat, processed food and snacks was associated with a higher risk of GDM.

## Discussion

This systematic review found a positive association between iron, processed meat and a low carbohydrate diet and GDM risk. Antioxidant nutrients, folic acid, fresh and dried fruits, vegetables, legumes and eggs were negatively associated with GDM. Generally, western dietary patterns increase GDM risk, and prudent dietary patterns or plant-based diets decrease the risk. It appears that a high intake of saturated fats at the expense of decreased carbohydrate intake is associated with an increased risk of GDM. Studies in both, humans and experimental animals, suggest that the adaptive phenotypic response to low-carbohydrate intake is insulin resistance [70]. These mechanisms, in sensitive organisms like pregnant women, are increased with diet exposure especially during this period [71]. However, these mechanisms need to be studied in greater depth.

As we have described, there is ample evidence considering diet an important factor in the prevention of GDM [72]. In this regard, national and international groups have identified preconception and pregnancy as key opportunities in the life course for health promotion and disease prevention [16, 61]. However, the current evidence about which nutrients, foods and diet characteristics are associated with the risk of developing GDM is based on a limited number of studies that are heterogeneous in design, sample size, exposure and outcome measures, and in the populations involved. Also, dietary components have been analysed in isolation, in food-groups or in dietary patterns.

Diet study from a dietary pattern approach is necessary because it makes it possible to study the associations between diet and the health-disease process, and to prevent incorrect interpretations of the results due to the complex interactions between the numerous components of the diet [15, 69]. Also, this approach is the most comprehensive and their results are the clearest for the development of health promotion actions due to their ability to capture the variability of food intake in a population influenced, in turn, by food availability and sociocultural factors. This could have better results and lower costs on health policies and clinical practice in developing countries [4–6, 9].

Most of the studies have been carried out in Asia, particularly in China and Iran, whose populations have lifestyles different from those of western countries, in addition to having genetic and cultural peculiarities. Likewise, in Africa, Oceania and Latin America the relationship between GDM and diet were poorly described. In addition, the GDM prevalence has been little described around the world too. Only in 2019, did the International Diabetes Federation (IDF) unify prevalence of hyperglycemia but not GDM prevalence [4]. However, the prevalence of GDM is estimated to increase [1, 3, 4].

As a limitation of the review, we found differences between the studies in the diagnostic criteria of GDM. Besides, the instruments for food data collection were validated but differently in each study because some of them used a food frequency questionnaire and others used a 24-hour dietary recall. Likewise, those kinds of instruments have measurement errors by memory bias in collection and the sample could have selection biases because most of the study populations were not drawn from a random sample, but from regions, cities or ethnic groups, which may limit the generalisability of the results. Although observational studies provide weaker evidence than other study designs, we focused on their analysis in order to synthesise evidence from feasible studies that could be conducted even in less socio-economically developed countries [73].

The results of this review are consistent with dietary recommendations for women of reproductive age or during pregnancy commonly indicated by healthcare professionals. Likewise, habitually there are recommendations for weight gain and symptoms treatments during pregnancy [74] and there is consensus on dietary recommendations for its treatment. However, there is no consensus on dietary recommendations for the prevention of GDM. We know the importance of proper nutrition as a pillar in the treatment of GDM, but it is necessary to highlight its importance in early pregnancy and even before pregnancy, in healthy women or women with associated risk factors and thus improve the quality of life of women and their offspring [75, 76].

As a conclusion, we consider that the physiology of pregnancy is homogeneous for all healthy women regardless of their place of residence. However, some will develop GDM, and some will not. Diet is considered one of the causes of GDM. However, there is no homogeneity in how people eat nor in how researchers assess diet. In this paper, we sought to build an integrated panorama of how habitual diet affects the risk of GDM as evaluated in different contextual conditions of the world.

## Supplementary Information

**Additional file 1. **The Newcastle-Ottawa quality assessment scale (NOS). **Supplementary Table 6.** Quality assessment of case-control studies on maternal dietary components and gestational diabetes. **Supplementary Table 7.** Quality assessment of cohort studies on maternal dietary components and gestational diabetes.

## Data Availability

Not applicable.

## References

[1] Mahajan A, Donovan LE, Vallee R, Yamamoto JM (2019). Evidence-based nutrition for gestational diabetes mellitus. Curr Diab Rep.

[2] Zhu Y, Zhang C (2016). Prevalence of gestational diabetes and risk of progression to type 2 diabetes: a global perspective. Curr Diab Rep.

[3] Faingold MC, Lamela C, Gheggi MS, Lapertosa S, Di Marco I (2009). Recommendations for pregnant women with diabetes: conclusions of the consensus meeting convened by the diabetes and pregnancy committee of the SAD. J Argentine Diab Soc.

[4] International Diabetes Federation (FID) (2021). IDF diabetes atlas.

[5] National Ministry of Health (2010). Guidelines for the diagnosis and treatment of hypertension in pregnancy.

[6] Simmons D (2019). GDM and nutrition-answered and unanswered questions-There's more work to do!. Nutrients..

[7] Vrachnis N, Antonakopoulos N, Iliodromiti Z, Dafopoulos K, Siristatidis C, Pappa KI (2012). Impact of maternal diabetes on epigenetic modifications leading to diseases in the offspring. Exp Diabetes Res.

[8] Farahvar S, Walfisch A, Sheiner E (2019). Gestational diabetes risk factors and long-term consequences for both mother and offspring: a literature review. Expert Rev Endocrinol Metab.

[9] Hassani Zadeh S, Boffetta P, Hosseinzadeh M (2020). Dietary patterns and risk of gestational diabetes mellitus: a systematic review and meta-analysis of cohort studies. Clin Nutr ESPEN.

[10] Shin D, Lee KW, Song WO (2015). Dietary patterns during pregnancy are associated with risk of gestational diabetes mellitus. Nutrients..

[11] Salzberg S, Alvariñas J, López G, Gorbán de Lapertosa S, Linari MA, Falcín E (2016). Gestational diabetes diagnosis and treatment guide. Rev ALAD.

[12] Donazar Ezcurra M, López del Burgo C, Bes RM (2017). Primary prevention of gestational diabetes mellitus through nutritional factors: a systematic review. BMC Pregnancy Childbirth.

[13] Raghavan R, Dreibelbis C, Kingshipp B (2019). Dietary patterns before and during pregnancy and maternal outcomes: a systematic review. Am J Clin Nutr.

[14] Jarman M, Mathe N, Ramazani F (2018). Dietary patterns prior to pregnancy and associations with pregnancy complications. Nutrients..

[15] Jacobs DR, Tapsell LC (2007). Food, not nutrients, is the fundamental unit in nutrition. Nutr Rev.

[16] Mijatovic-Vukas J, Capling L, Cheng S, Stamatakis E, Louie J, Cheung NW (2018). Associations of diet and physical activity with risk for gestational diabetes mellitus: a systematic review and meta-analysis. Nutrients..

[17] Mousa A, Naqash A, Lim S (2019). Macronutrient and micronutrient intake during pregnancy: an overview of recent evidence. Nutrients..

[18] Wells GA, Shea B, O’Connell D (2014). The Newcastle-Ottawa scale (NOS) for assessing the quality of nonrandomised studies in meta-analyses [article online].

[19] Asadi M, Shahzeidi M, Najarzadeh A, Hashemi Yusefabad H, Mansoori A (2019). The relationship between pre-pregnancy dietary patterns adherence and risk of gestational diabetes mellitus in Iran: a case–control study. Nutr Diet.

[20] Sedaghat F, Akhoondan M, Ehteshami M, Aghamohammadi V, Ghanei N, Mirmiran P (2017). Maternal dietary patterns and gestational diabetes risk: a case-control study. J Diabetes Res.

[21] Lamyian M, Hosseinpour-Niazi S, Mirmiran P, Moghaddam Banaem L, Goshtasebi A, Azizi F (2017). Pre-pregnancy fast food consumption is associated with gestational diabetes mellitus among Tehranian women. Nutrients..

[22] Zamani B, Milajerdi A, Tehrani H, Bellissimo N, Brett NR, Azadbakht L (2019). Association of a plant-based dietary pattern in relation to gestational diabetes mellitus. Nutr Diet.

[23] Zareei S, Homayounfar R, Naghizadeh MM, Ehrampoush E, Rahimi M (2018). Dietary pattern in pregnancy and risk of gestational diabetes mellitus (GDM). Diabetes Metab Syndr.

[24] Du HY, Jiang H, Karmin O, Chen B, Xu LJ, Liu SP (2017). Association of dietary pattern during pregnancy and gestational diabetes mellitus: a prospective cohort study in northern China. Biomed Environ Sci.

[25] Chen Q, Wu W, Yang H, Zhang P, Feng Y, Wang K (2020). A vegetable dietary pattern is associated with lowered risk of gestational diabetes mellitus in Chinese women. Diabetes Metab J.

[26] Zhou X (2018). Maternal dietary pattern characterised by high protein and low carbohydrate intake in pregnancy is associated with a higher risk of gestational diabetes mellitus in Chinese women: a prospective cohort study. Br J Nutr.

[27] Dong H, Sun H, Cai C, Pang X, Bai D, Lan X (2021). A low-carbohydrate dietary pattern characterised by high animal fat and protein during the first trimester is associated with an increased risk of gestational diabetes mellitus in Chinese women: a prospective cohort study. Br J Nutr.

[28] Chen Q, Feng Y, Yang H, Wu W, Zhang P, Wang K (2019). A vitamin pattern diet is associated with decreased risk of gestational diabetes mellitus in Chinese women: results from a case control study in Taiyuan, China. J Diabetes Res.

[29] Liu C, Zhong C, Chen R, Zhou X, Wu J, Han J (2020). Higher dietary vitamin C intake is associated with a lower risk of gestational diabetes mellitus: a longitudinal cohort study. Clin Nutr.

[30] Saraf-Bank S, Tehrani H, Haghighatdoost F, Moosavian SP, Azadbakht L (2018). The acidity of early pregnancy diet and risk of gestational diabetes mellitus. Clin Nutr.

[31] Parast VM, Paknahad Z (2017). Antioxidant status and risk of gestational diabetes mellitus: a case-control study. Clin Nutr Res.

[32] Gao Q, Zhong C, Zhou X, Chen R, Xiong T, Hong M (2019). The association between intake of dietary lycopene and other carotenoids and gestational diabetes mellitus risk during mid-trimester: a cross-sectional study. Br J Nutr.

[33] Kyozuka H, Murata T, Fukuda T, Yamaguchi A, Kanno A, Yasuda S (2021). Effect of preconception selenium intake on the risk for gestational diabetes: the Japan environment and Children's study. Antioxidants (Basel).

[34] Daneshzad E, Tehrani H, Bellissimo N, Azadbakht L (2020). Dietary Total antioxidant capacity and gestational diabetes mellitus: a case-control study. Oxidative Med Cell Longev.

[35] Aljanahi A, Hadhiah H, Al-Nasr W, Abuzaid O, Al Qahtani N, Sebastian T (2020). The effect of dietary intake of vitamin D on gestational diabetes mellitus. Nutr Metab Insights.

[36] Li H, Xie S, Zhang X, Xia Y, Zhang Y, Wang L (2021). Mid-pregnancy consumption of fruit, vegetable and fruit juice and the risk of gestational diabetes mellitus: a correlation study. Clin Nutr ESPEN.

[37] Yong HY, Mohd Shariff Z, Mohd Yusof BN (2021). Beverage intake and the risk of gestational diabetes mellitus: the SECOST. Nutrients..

[38] Liu YH, Lu LP, Yi MH (2022). Study on the correlation between homocysteine-related dietary patterns and gestational diabetes mellitus:a reduced-rank regression analysis study. BMC Pregnancy Childbirth.

[39] Wang H, Huang L, Lin L (2021). The overall plant-based diet index during pregnancy and risk of gestational diabetes mellitus: a prospective cohort study in China. Br J Nutr.

[40] Zhang X, Gong Y, Della Corte K (2021). Relevance of dietary glycemic index, glycemic load and fiber intake before and during pregnancy for the risk of gestational diabetes mellitus and maternal glucose homeostasis. Clin Nutr.

[41] Tryggvadottir EA, Medek H, Birgisdottir BE, Geirsson RT, Gunnarsdottir I (2016). Association between healthy maternal dietary pattern and risk for gestational diabetes mellitus. Eur J Clin Nutr.

[42] Kozlowska A, Jagielska AM, Okreglicka KM, Dabrowski F, Kanecki K, Nitsch-Osuch A (2018). Dietary vitamin and mineral intakes in a sample of pregnant women with either gestational diabetes or type 1 diabetes mellitus, assessed in comparison with polish nutritional guidelines. Ginekol Pol.

[43] Bartáková V, Kuricová K, Zlámal F, Bělobrádková J, Kaňková K (2018). Differences in food intake and genetic variability in taste receptors between Czech pregnant women with and without gestational diabetes mellitus. Eur J Nutr.

[44] Donazar-Ezcurra M, Lopez-Del Burgo C, Martinez-Gonzalez MA, Basterra-Gortari FJ, de Irala J, Bes-Rastrollo M (2017). Pre-pregnancy adherences to empirically derived dietary patterns and gestational diabetes risk in a Mediterranean cohort: the Seguimiento Universidad de Navarra (SUN) project. Br J Nutr.

[45] Marí-Sanchis A, Díaz-Jurado G, Basterra-Gortari FJ, de la Fuente-Arrillaga C, Martínez-González MA, Bes-Rastrollo M (2018). Association between pre-pregnancy consumption of meat, iron intake, and the risk of gestational diabetes: the SUN project. Eur J Nutr.

[46] Petry CJ, Ong KK, Hughes IA, Acerini CL, Dunger DB (2019). Temporal trends in maternal food intake frequencies and associations with gestational diabetes: the Cambridge baby growth study. Nutrients..

[47] Nicolì F, Prete A, Citro F (2021). Use of non-nutritive-sweetened soft drink and risk of gestational diabetes. Diabetes Res Clin Pract.

[48] Yuste Gómez A, Ramos Álvarez MDP, Bartha JL (2022). Influence of diet and lifestyle on the development of gestational diabetes mellitus and on perinatal results. Nutrients..

[49] Bao W, Song Y, Bertrand KA, Tobias DK, Olsen SF, Chavarro JE (2017). Prepregnancy habitual intake of vitamin D from diet and supplements in relation to risk of gestational diabetes mellitus: a prospective cohort study. J Diabetes.

[50] Li M, Li S, Chavarro JE, Gaskins AJ, Ley SH, Hinkle SN (2019). Pre pregnancy habitual intakes of Total, supplemental, and food Folate and risk of gestational diabetes mellitus: a prospective cohort study. Diabetes Care.

[51] Osorio-Yáñez C, Gelaye B, Qiu C, Bao W, Cardenas A, Enquobahrie DA, Williams MA. Maternal intake of fried foods and risk of gestational diabetes mellitus. Ann Epidemiol. 2017;27(6):384–390.e1. 10.1016/j.annepidem.2017.05.006 PMC5578760 28641758

[52] Darling AM, Mitchell AA, Werler MM (2016). Preconceptional iron intake and gestational diabetes mellitus. Int J Environ Res Public Health.

[53] Chen Z, Qian F, Liu G (2021). Prepregnancy plant-based diets and the risk of gestational diabetes mellitus: a prospective cohort study of 14,926 women. Am J Clin Nutr.

[54] Lindsay KL, Milone GF, Grobman WA (2022). Periconceptional diet quality is associated with gestational diabetes risk and glucose concentrations among nulliparous gravidas. Front Endocrinol (Lausanne).

[55] Looman M, Schoenaker DAJM, Soedamah-Muthu SS, Mishra GD, Geelen A, Feskens EJM (2019). Pre-pregnancy dietary micronutrient adequacy is associated with lower risk of developing gestational diabetes in Australian women. Nutr Res.

[56] Mahjoub F, Ben Jemaa H, Ben Sabeh F, Ben Amor N, Gamoudi A, Jamoussi H (2021). Impact of nutrients and Mediterranean diet on the occurrence of gestational diabetes. Libyan J Med.

[57] Sartorelli DS, Zuccolotto DCC, Crivellenti LC, Franco LJ (2019). Dietary patterns during pregnancy derived by reduced-rank regression and their association with gestational diabetes mellitus. Nutrition..

[58] Balbi MA, Crivellenti LC, Zuccolotto DCC, Franco LJ, Sartorelli DS (2019). The relationship of flavonoid intake during pregnancy with excess body weight and gestational diabetes mellitus. Arch Endocrinol Metab.

[59] Nascimento GR, Alves LV, Fonseca CL, Figueiroa JN, Alves JG (2016). Dietary patterns and gestational diabetes mellitus in a low income pregnant women population in Brazil . A cohort study. Arch Latinoam Nutr.

[60] Barbieri P, Nunes JC, Torres AG, Nishimura RY, Zuccolotto DC, Crivellenti LC (2016). Indices of dietary fat quality during midpregnancy is associated with gestational diabetes. Nutrition..

[61] Hill B, Skouteris H, Teede HJ, Bailey C, Baxter J-AB, Bergmeier HJ (2019). Health in preconception, pregnancy and postpartum global Alliance: international network preconception research priorities for the prevention of maternal obesity and related pregnancy and long-term complications. Journal of. Clinical Medicine.

[62] National Ministry of Health (2012). Nutrition and pregnancy. Recommendations on nutrition for health teams -National Directorate of maternity and childhood.

[63] Kim SY, England L, Wilson HG, Bish C, Satten GA, Dietz P (2010). Percentage of gestational diabetes mellitus attributable to overweight and obesity. Am J Public Health.

[64] Anderson AS (2001). Symposium on ‘nutritional adaptation to pregnancy and lactation’. Pregnancy as a time for dietary change?. Proc Nutr Soc.

[65] Karamanos B, Thanopoulou A, Anastasiou E, Assaad-Khalil S, Albache N, Bachaoui M (2014). Relation of the Mediterranean diet with the incidence of gestational diabetes. Eur J Clin Nutr.

[66] Sartorelli SD, Crivellenti CL, Zuccolotto DCC, Franco LJ (2019). Relationship between minimally and ultra-processed food intake during pregnancy with obesity and gestational diabetes mellitus. Cad Saúde Pública.

[67] Edefonti V, Randi G, La Vecchia C (2009). Dietary patterns and breast cancer: a review with focus on methodological issues. Nutr Rev.

[68] Kac G, Sichieri R, Petrucci Gigante D (2007). Epidemiologia Nutricional.

[69] Paknahad Z, Fallah A, Moravejolahkami AR (2019). Maternal dietary patterns and their association with pregnancy outcomes. Clin Nutr Res.

[70] Brand Miller JC, Colagiuri S (1994). The carnivore connection: dietary carbohydrate in the evolution of NIDDM. Diabetologia..

[71] Butte NF (2000). Carbohydrate and lipid metabolism in pregnancy: normal compared with gestational diabetes mellitus. Am J Clin Nutr.

[72] Martínez VLE (2008). Programación fetal de enfermedades expresadas en la etapa adulta. Med Univer.

[73] Horta BL, Wehrmeister FC. ¿Cuál es la importancia de las cohortes y los análisis del ciclo vital? Cad. Saúde Pública. 2017;33(3):e00035717. 10.1590/0102-311X00035717 28380141

[74] Rasmussen KM, Yaktine AL (2009). Committee to reexamine IOM pregnancy weight guidelines.

[75] Parekh N, Zizza C (2013). Life course epidemiology in nutrition and chronic disease research: a timely discussion. Adv Nutr.

[76] Phillips CM (2017). Metabolically healthy obesity across the life course: epidemiology, determinants, and implications. Ann N Y Acad Sci.

